# Nasal Nitric Oxide in Chronic Rhinosinusitis with or without Nasal Polyps: A Systematic Review with Meta-Analysis

**DOI:** 10.3390/jcm9010200

**Published:** 2020-01-11

**Authors:** Pasquale Ambrosino, Antonio Molino, Giorgio Alfredo Spedicato, Paolo Parrella, Roberto Formisano, Andrea Motta, Matteo Nicola Dario Di Minno, Mauro Maniscalco

**Affiliations:** 1 Cardio-Pulmonary Rehabilitation Dept, Istituti Clinici Scientifici Maugeri IRCCS, 82037 Telese Terme (BN), Italy; pasquale.ambrosino@icsmaugeri.it (P.A.); paolo.parrella@icsmaugeri.it (P.P.); roberto.formisano@icsmaugeri.it (R.F.); 2 Respiratory Division, Department of Respiratory Medicine, Federico II University, 80131 Naples, Italy; molinotonio@libero.it; 3 Unipol Group, 40128 Bologna, Italy; 4 Institute of Biomolecular Chemistry, National Research Council, 80078 Pozzuoli (Naples), Italy; andrea.motta@icb.cnr.it; 5 Department of Translational Medical Sciences, Federico II University, 80131 Naples, Italy; matteo.diminno@unina.it; 6 Istituti Clinici Scientifici Maugeri IRCCS, Via Maugeri 4, 27100 Pavia, Italy

**Keywords:** chronic rhinosinusitis, nasal polyps, biomarkers, outcome, allergy, asthma, disability

## Abstract

Background and Aims: There has been a recent growing interest in the role of nasal nitric oxide (nNO) as a biomarker for osteomeatal complex obstruction in paranasal sinus diseases. By using meta-analysis, we systematically reviewed the literature to establish the possible link between nNO concentration and chronic rhinosinusitis with nasal polyps (CRSwNP) or without (CRSsNP). Methods: We systematically searched the EMBASE, PubMed, Scopus, and Web of Science databases for related studies. Differences between controls and cases were reported as standardized mean difference (SMD), with 95% confidence intervals (95% CI), using the random-effects method. Results: We selected 23 articles for the final analysis: 15 with data on 461 CRSwNP patients and 384 healthy controls, 10 with data on 183 CRSsNP patients and 260 controls, and 14 studies on 372 CRSwNP and 297 CRSsNP patients. CRSwNP patients showed significantly lower nNO values when compared to both healthy controls (SMD: −1.495; 95% CI: −2.135, −0.854; *p* < 0.0001) and CRSsNP patients (SMD: −1.448; 95% CI: −2.046, −0.850; *p* < 0.0001). Sensitivity and subgroup analyses confirmed the results, which were further refined by regression models. They showed that an increasing aspiration flow is related to a greater difference in nNO levels between cases and control subjects. We also documented lower nNO levels in CRSsNP patients with respect to controls (SMD: −0.696; 95% CI: −1.189, −0.202; *p* = 0.006), being this result no longer significant when excluding patients in therapy with intranasal corticosteroids. As shown by regression models, the increased Lund–Mackay score indicates a high effect size. Conclusions: nNO levels are significantly lower in CRSwNP, especially when using higher aspiration flows. Additional studies are needed to define one single standardized method and normal reference values for nNO.

## 1. Introduction

The role of nitric oxide (NO) in respiratory medicine has received increasing attention in the last years. It was first described as a simple vasodilator [1], then several functions were assigned to NO in the airways, where it acts as bronchodilator, neurotransmitter, antimicrobial, antitumor, and mucociliary regulator [2,3,4]. Both in-vivo and in-vitro data showed that in chronic inflammatory pulmonary diseases, levels of NO in exhaled air are mostly augmented [5]. Consequently, the measurement of fractional exhaled NO (FeNO) has become an essential tool for monitoring asthma and other pulmonary diseases [6,7].

Regarding the nasal route, the paranasal sinuses and (to a lesser extent) the nasal mucosa are responsible for the production of NO [8]. Considering that NO can be easily measured also in the upper respiratory tract, some authors hypothesized that nasal NO (nNO) could become an objective support to diagnose and monitor obstruction and inflammation in the upper airways [9,10] with limited invasiveness. Although the American Thoracic Society and the European Respiratory Society have approved a highly standardized method for measuring FeNO [11,12,13], no single standardized procedure for nNO estimation was defined. As a matter of fact, several techniques have been used so far to evaluate nNO levels, with different sampling methods and different ways to exclude the lower respiratory tract [14].

Regardless of the method used, over the last few decades, it was reported that nNO is a useful screening test for primary ciliary dyskinesia (PCD), since nNO is markedly reduced in this condition with all available techniques [15]. More recently, the association between other diseases of the upper airways and nNO levels has been evaluated. In particular, lower nNO levels were observed in chronic rhinosinusitis with nasal polyps (CRSwNP) or without (CRSsNP) [16,17]. It is still uncertain whether the low NO levels detected in chronic rhinosinusitis result from a reduced maxillary NO production, or are rather mainly due to an obstruction of sinus ostia. Moreover, other studies hypothesized a role for nNO in discriminating the presence and absence of polyposis when in the presence of chronic rhinosinusitis, being nNO considerably reduced in CRSwNP with respect to CRSsNP [18,19]. Although doubts have been cast on these results [20,21], no meta-analyses evaluating published data presently exist.

In the present study, we performed a methodical review and meta-analysis of data assessing the association between paranasal sinus inflammatory diseases (CRSwNP and CRSsNP) and nNO levels. Furthermore, we employed some meta-regression models to estimate the impact of some clinical and demographic data on these outcomes.

## 2. Methods

We prospectively developed a protocol for this analysis, specifying the objectives, the selection criteria, the method to evaluate study quality, the outcomes, and the statistical methods.

### 2.1. Search Strategy

We systematically searched the electronic databases (EMBASE, PubMed, Scopus, and Web of Science) consistently with the Preferred Reporting Items for Systematic Reviews and Meta-Analyses (PRISMA) guidelines [22]. All possible combinations of the following terms were used: chronic rhinosinusitis, nasal nitric oxide, nasal polyps, and nasal polyposis. We performed the last search on 16 October 2019, applying no constraint on the publication language in the search strategy.

**Figure 1:**
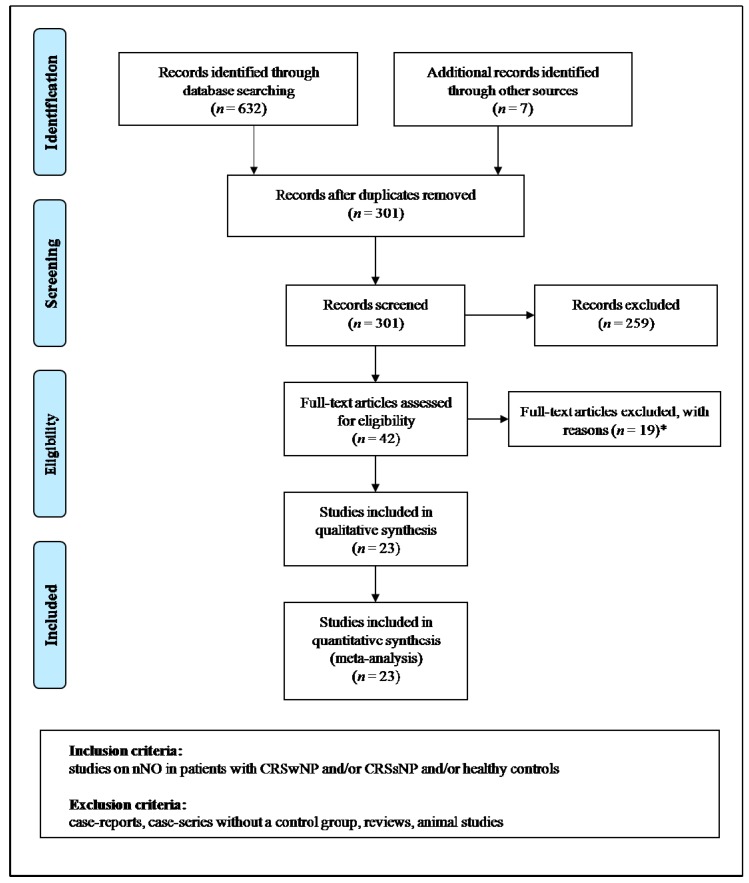
Preferred Reporting Items for Systematic Reviews and Meta-Analyses (PRISMA) for study selection. *Two studies on chronic rhinosinusitis (CRS) with no clear report of the number of patients with concomitant nasal polyps (NP), 13 studies without control group, two studies on fractional exhaled nitric oxide (FeNO), and two studies only reporting *p* values and/or means without standard deviations or standard errors. CRSwNP: chronic rhinosinusitis with nasal polyps; CRSsNP: chronic rhinosinusitis without nasal polyps.

Moreover, the retrieved studies were manually revised. For missing data, we contacted the authors to obtain original data. P.A. and M.M., two of the authors, independently analyzed the found articles and carried out data extraction. When P.A. and M.M. disagreed, a third investigator (A.M.) was consulted, and discrepancies were resolved by consensus. Selection results presented a high inter-reader agreement (κ = 1.00) and were described in accordance with PRISMA flowchart (Figure 1).

### 2.2. Data Extraction and Quality Assessment

In agreement with the established protocol, we considered all studies evaluating the level of nNO in CRSwNP and/or CRSsNP patients and/or healthy controls. We excluded case reports, case series devoid of a control group, reviews, and studies involving animals. Abstracts and citations from scientific conferences were also included. Inclusion in the analysis required presence in the study of nNO values (mean with standard deviation or standard error) for CRSwNP and/or CRSsNP patients, and/or healthy subjects. The comprised studies were categorized as having a prospective or retrospective design.

In each study, we considered sample size, major clinical and demographic variables, nNO data in CRSwNP and/or CRSsNP patients, and/or healthy subjects. Chronic rhinosinusitis data with an unclear number of subjects with concomitant polyposis were excluded. Table S1 describes methods and devices used for nNO assessment in the selected studies.

Given the features of the considered studies, we used the Newcastle–Ottawa Scale (NOS) to evaluate the methodological quality of each study. It was explicitly developed to estimate the quality of non-randomized observational studies [23]. The scoring system comprehends three major domains: selection (4 items), comparability (1 item), and exposure (3 items). A maximum score of 1 is graded for each item, except those related to comparability, which allows for 2. Total scores are calculated by adding the score for each item, with a resulting 0–9 score range, with the better methodological quality identified by a higher score. Table S2 reports the calculation of the NOS quality assessment.

### 2.3. Statistical Analysis and Assessment of the Risk of Bias

Comprehensive Meta-analysis (Version 2 (2005), Biostat, Englewood, NJ, USA) was used for statistical analysis. Differences between cases and controls were expressed as standardized mean difference (SMD) with a pertinent 95% confidence interval (95% CI). A different variance of the estimator among studies was assumed for the analyses.

The total effect was tested by means of Z scores, and significance was considered at *p* < 0.05. Statistical heterogeneity between studies was evaluated with chi-square Cochran’s Q test and I^2^ statistics, which measure the inconsistency across the results of the studies, and define the proportion of total variation in the estimates of the studies that are related to heterogeneity rather than sampling error. In detail, I^2^ = 0% indicates no heterogeneity; 25%, low; 25–50%, moderate; and >50%, high heterogeneity [24].

Publication bias was graphically represented by funnel plots of the effect size (SMD) vs. precision (1/standard error of the SMD) for studies evaluating nNO in cases and in control subjects. We visually examined the asymmetry of funnel plots to take care of possible small-study effect. Egger’s and Begg and Mazumdar tests were used to estimate publication bias, over and above any subjective evaluation. A *p* < 0.10 was considered statistically significant [25]. Moreover, the Duval and Tweedie’s trim and fill analysis was used to estimate an adjusted effect size after trimming and imputing studies [26].

In order to be as conservative as possible, we used the random-effect method to consider the variability among comprised studies.

### 2.4. Sensitivity Analyses

To investigate potential sources of heterogeneity, we repeated analyses by including only “high quality” studies according to NOS (i.e., NOS ≥ median value found among considered studies). Moreover, to prevent the risk of data overlap, a sensitivity analysis was carried out after eliminating studies including identical enrollment Centers, enrolling subjects in the same period in common with other considered studies.

Given the possible impact of intranasal corticosteroids on nNO levels, distinct analyses of studies explicitly avoiding the use of local steroids were planned.

### 2.5. Subgroup Analyses

Subgroup analyses were carried out for studies measuring nNO with different techniques (nasal aspiration and nasal exhalation). We also planned to execute further subgroup analyses after studies’ stratification were done according to design (prospective and retrospective).

### 2.6. Meta-Regression Analyses

We hypothesized that discrepancies among considered studies may be due to demographic variables (mean age, male gender) and clinical data, such as atopic status, simultaneous presence of asthma, smoking habit, radiographic extent of disease (Lund–Mackay computed tomography (CT) score), subjective evaluation of nasal symptoms (Sino-Nasal Outcome Test-22 (SNOT-22)), pulmonary function values (forced expiratory volume in 1 second (FEV_1_), forced vital capacity (FVC), FEV_1_/FVC), and transnasal aspiration flow during nNO assessment. To assess potential effects of the above variables in describing differences observed across studies, we planned meta-regression analyses after employing a regression model with changes in nNO as dependent variables (*y*) and the above-indicated covariates as independent variables (*x*).

## 3. Results

Once duplicates were excluded, our exploration retrieved 301 papers. From them, we eliminated 174 articles for the reason that they were unrelated to the topic of the meta-analysis after evaluating the title and/or the abstract, and some other 85 because they were case reports/comments/reviews, or did not include related data. For one study, we could not retrieve the full-length version but the data were included in the abstract. Finally, 19 papers were not included after a complete evaluation of the text. Therefore, 23 papers were considered for the conclusive analysis [16,17,18,19,20,21,27,28,29,30,31,32,33,34,35,36,37,38,39,40,41,42,43] (Figure 1). Of these, seven studies [16,31,32,36,37,38,40] provided data on CRSwNP patients and healthy controls, two studies [17,27] on CRSsNP and healthy subjects, six studies [18,19,20,41,42,43] on CRSwNP and CRSsNP patients, while eight studies [21,28,29,30,33,34,35,39] evaluated both clinical settings (CRSwNP and CRSsNP) and healthy subjects.

Overall, 15 studies (18 data sets) analyzed data from 461 CRSwNP patients and 384 healthy controls, 10 studies from 183 CRSsNP patients and 260 controls, and 14 studies (15 data sets) from 372 CRSwNP and 297 CRSsNP patients. All studies had a prospective design.

### 3.1. Study Characteristics

The main demographic and clinical data of the enrolled subjects are reported in Table 1. The number of CRSwNP patients ranged between 6 and 57, the mean age was between 33.4 and 57.9 years, and 50–77.8% was the occurrence of male gender. Active smoking was reported by 0–33.3% of subjects. The prevalence of atopy or asthma ranged from 0% to 100% of patients. The use of local steroids was an exclusion criterion in most included studies, being reported by 0–100% of CRSwNP patients. Mean Lund–Mackay score varied from 10.8 to 16.9, mean SNOT-22 score from 28.2 to 42.7, and mean FEV_1_ values from 80% to 101.1% predicted. Only one study [33] reported data on mean FEV_1_/FVC (92.0), while no study reported FVC values. 

Studies reporting on CRSsNP enrolled from 7 to 46 patients, with a mean age of 42 to 56 years, and a 13–79.4% prevalence of male gender. Atopy/asthma was disclosed by 0–100% of CRSsNP subjects, smoking by 0–30.7%, and the use of intranasal corticosteroids by 0–100%, with the use of local steroids being an exclusion criterion in most cases. Mean values of the Lund–Mackay score varied from 3 to 11.8, while the SNOT-22 score ranged from 25.2 to 50.7. Only one study [33] reported values of FEV_1_ (81% predicted) and FEV_1_/FVC (92.0). No study reported FVC values.

Two studies [16,28] provided distinct data for CRSwNP subjects with/without allergy, while two [36,43] for patients undergoing medical or surgical therapy for nasal polyps or chronic rhinosinusitis. In these cases, different groups were evaluated as different data sets. Most included studies [16,17,18,20,21,27,28,30,31,32,33,34,35,37,38,40,42,43] evaluated nNO by nasal aspiration method, while only five studies [19,29,36,39,41] used nasal exhalation.

### 3.2. nNO in CRSwNP and Healthy Controls

**Figure 2:**
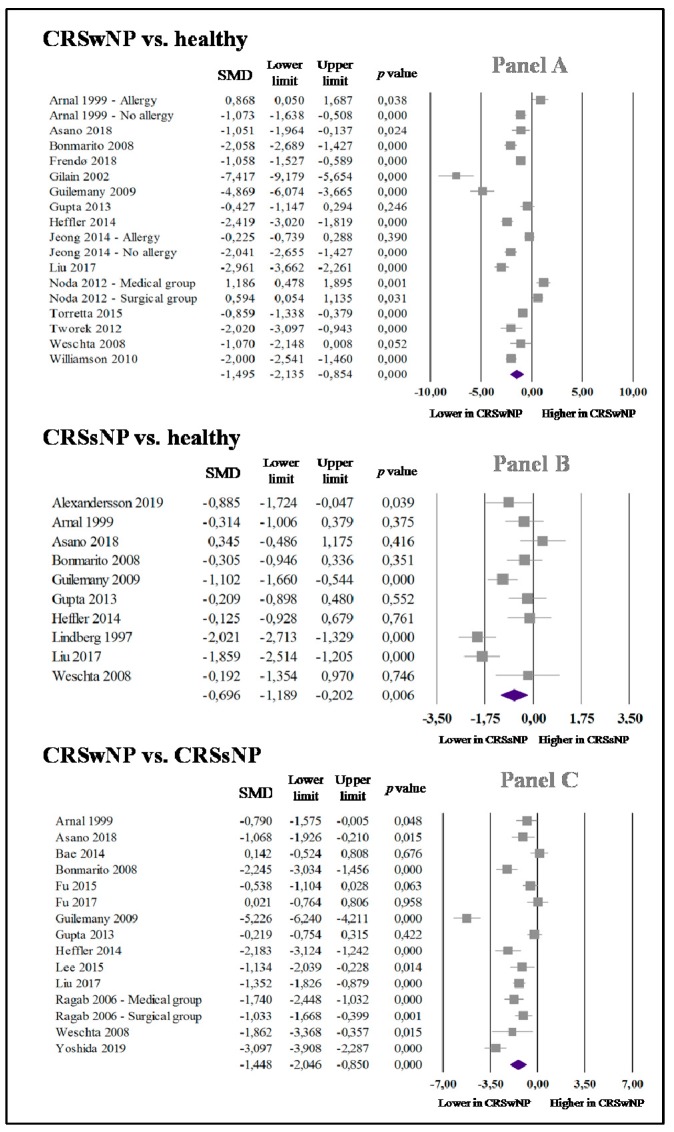
Forest plot of the standardized mean difference (SMD) in nasal nitric oxide (nNO) levels among cases and control subjects. Panel A: nNO in patients with chronic rhinosinusitis with nasal polyps (CRSwNP) and healthy controls; Panel B: nNO in patients with chronic rhinosinusitis without nasal polyps (CRSsNP) and healthy controls; Panel C: nNO in CRSwNP and CRSsNP patients.

In 15 studies (18 data sets) [16,21,28,29,30,31,32,33,34,35,36,37,38,39,40], the 461 CRSwNP subjects presented significantly reduced nNO values with respect to the 384 healthy subjects (SMD: −1.495; 95% CI: −2.135, −0.854; *p* < 0.0001, Figure 2 (Panel A)). Statistical significance (I^2^ = 93.9%; *p* < 0.0001) was found for studies’ heterogeneity, and it was not reduced after individually eliminating each study.

### 3.3. nNO in CRSsNP and Healthy Subjects

Ten studies [17,21,27,28,29,30,33,34,35,39] on 183 cases and 260 controls indicated that CRSsNP subjects exhibit considerably reduced levels of nNO in comparison to controls (SMD: −0.696; 95% CI: −1.189, −0.202; *p* = 0.006, Figure 2 (Panel B)). The reports presented a statistically significant high heterogeneity (I^2^ = 78.2%; *p* < 0.0001); it was not reduced after individually eliminating each study.

### 3.4. nNO in CRSwNP and CRSsNP

Fourteen studies (15 data sets) [18,19,20,21,28,29,30,33,34,35,39,41,42,43] showed that 372 CRSwNP patients had significantly lower nNO values than 297 subjects with CRSsNP (SMD: −1.448; 95% CI: −2.046, −0.850; *p* < 0.0001, Figure 2 (Panel C)). A statistically significant high heterogeneity (I^2^ = 90.0%; *p* < 0.0001) was found, and it was not reduced after individually eliminating each study.

### 3.5. Publication Bias

It is well known that publication bias is able to alter meta-analyses’ results, thus funnel plot analysis was used to evaluate this potential bias.

Examination of funnel plots of effect size vs. precision hints at no bias and small-study effect for studies evaluating nNO in CRSwNP patients and in healthy controls (Figure S1), and for those evaluating this biomarker in CRSsNP and healthy subjects (Figure S2). No relevant publication bias was also confirmed by Egger’s and Begg and Mazumdar tests. Furthermore, the Duval and Tweedie’s trim and fill analyses indicated that all results were validated after trimming and imputing analyses.

When considering studies comparing CRSwNP and CRSsNP patients, funnel plot examination put forward the presence of possible publication bias and of small-study effect (Figure S3), substantiated by the Egger’s (*p* = 0.083) and Begg and Mazumdar tests (*p* = 0.023, based on continuity-corrected normal approximation). Remarkably, the adjusted effect size obtained by using the Duval and Tweedie’s trim and fill analysis suggested that, after trimming and imputing studies (*n* = 4), significantly lower nNO levels were confirmed in CRSwNP with respect to subjects with CRSsNP (SMD: −1.954; 95% CI: −2.641, −1.268).

### 3.6. Sensitivity Analyses

We found a median value of 6 for NOS quality assessment (Table S2). Consequently, the analyses were carried out again by considering only the 18 “high quality” papers with NOS ≥ 6 [16,18,19,20,21,27,28,29,30,31,33,34,35,38,39,40,42,43]. For three studies [32,37,41], relevant data were derived from the abstract, thus no quality evaluation could be achieved. Interestingly, without “low quality” studies [17,36] and those with only the abstract available [32,37,41], we substantially confirmed all results (Table 2 (Panel A)). Comparable results were also obtained, eliminating one study [20] possibly considering the same population involved in a different study [42] (Table 2 (Panel B)).

Given the potential influence of local steroids on nNO levels, a sensitivity analysis was planned for studies specifically excluding patients taking intranasal corticosteroids [16,17,19,29,30,31,34,35,36,38,39,40]. As a result, CRSwNP patients confirmed significantly lower nNO levels as compared to healthy subjects, with an even higher effect size when compared to CRSsNP patients (SMD: −1.953; 95% CI: −2.588, −1.318; *p* < 0.0001, I^2^ = 90.0%; *p* < 0.0001). In contrast, when excluding the use of local steroids, the difference between CRSsNP and healthy subjects was no longer significant (Table 2 (Panel C)). Separate analyses of patients following a local-steroid therapy were not planned because only very few studies (*n* = 3) included or specifically enrolled subjects treated with such medications for nasal symptoms.

### 3.7. Subgroup Analyses

Separate analyses were performed for studies measuring nNO with different techniques. Interestingly, all results were substantially confirmed when considering studies [16,17,18,20,21,27,28,30,31,32,33,34,35,37,38,40,42,43] evaluating nNO by nasal aspiration (Table 3 (Panel A)) but not when analyzing five studies [19,29,36,39,41] on nasal exhalation (Table 3 (Panel B)). Since all considered studies were prospective, we did not conduct a subgroup analysis following the study design (prospective or retrospective).

### 3.8. Meta-Regression Analyses

**Figure 3:**
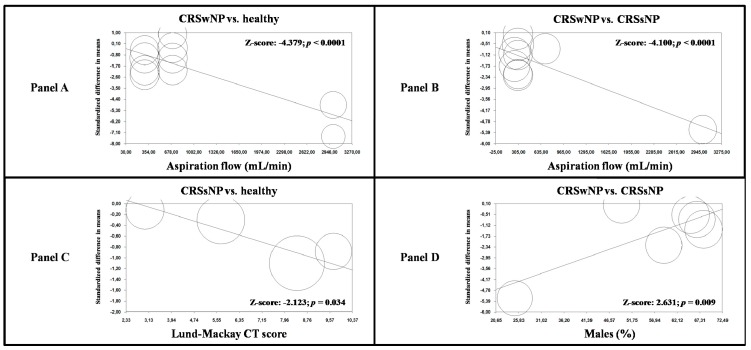
Meta-regression analyses. (Panel **A**): impact of aspiration flow on nNO in CRSwNP patients and healthy controls; (Panel **B**): impact of aspiration flow on nNO in CRSwNP and CRSsNP patients; (Panel **C**): impact of Lund–Mackay CT score in CRSsNP patients and healthy controls; (Panel **D**): impact of male gender on nNO in CRSwNP and CRSsNP patients. nNO: nasal nitric oxide; CRSwNP: chronic rhinosinusitis with nasal polyps; CRSsNP: chronic rhinosinusitis without nasal polyps; CT: computed tomography.

Regression models indicated that the aspiration flow during nNO assessment significantly impacted nNO levels when comparing CRSwNP and healthy subjects (Z-score: −4.379; *p* < 0.0001, Figure 3A), and for studies on CRSwNP and CRSsNP patients (Z-score: −4.100, *p* < 0.0001, Figure 3B). Moreover, an increasing Lund–Mackay CT score was connected with an increased difference in nNO levels between CRSsNP and healthy subjects (Z-score: −2.123; *p* = 0.034, Figure 3C).

Finally, a growing male percentage was related to a low effect size for studies comparing CRSwNP and CRSsNP patients (Z-score: 2.361; *p* = 0.009, Figure 3D). No other demographic and clinical variables affected the observed results (Table S3).

## 4. Discussion

In this systematic review with meta-analysis, we have shown that CRSwNP patients exhibit significantly lower nNO values as compared to both CRSsNP and healthy subjects. These results are substantially confirmed by appropriate sensitivity and subgroup analyses, and further refined by regression models, showing that an increasing aspiration flow during nNO assessment is related to an increased difference in nNO levels between cases and control subjects.

The results of the meta-analysis also documented decreased nNO values in CRSsNP with respect to healthy subjects. Concerning sensitivity analysis, this result was no longer confirmed when excluding CRSsNP patients taking intranasal corticosteroids. Moreover, regression models indicated that a growing Lund–Mackay CT score is related to a higher effect size.

Overall, these findings suggest that nNO could be effectively and routinely used as a marker of obstruction of the osteomeatal complex in patients with paranasal sinus inflammatory disease, just like FeNO is considered an established and recognized means to diagnose and monitor asthma [6,7]. In fact, we found that CRSwNP patients exhibit significantly decreased nNO values in comparison with CRSsNP, thus suggesting the potential role of nNO in indicating the presence/absence of polyposis in patients with chronic rhinosinusitis. This is in line with our meta-regression analyses, indicating that an increasing nasal CT score relates to a higher difference in nNO levels between CRSsNP and healthy subjects.

However, the low nNO levels found in patients with chronic rhinosinusitis can also be explained through other mechanisms, such as the nasal obstruction resulting from mucosal swelling and presence or absence of nasal secretions [44] or the damage of the NO-producing sinus mucosa by an increased synthesis of cytotoxic agents in chronic inflammation [4]. Moreover, reduced expression of the inducible isoform of NO synthase (iNO-synthase) caused by some inflammatory cytokines (IL-4, IL-6, and TGF-β) has been found in the sinus mucosa of chronic rhinosinusitis patients [4]. More recently, it has been suggested that an increased arginase activity in patients with chronic rhinosinusitis may decrease NO levels by means of reduced availability of L-arginine, the main NO precursor [45]. This might conflict with a great amount of other experimental and clinical evidences thus far reported, which show that inflammation is able to modulate iNO-synthase expression, thus leading to higher NO production in patients with inflammation of the respiratory tract [46,47,48,49]. It should be noted that our results on nNO in CRSsNP subjects compared to healthy controls were not confirmed when considering patients without ongoing therapy with intranasal corticosteroids. Accordingly, when excluding the use of local steroids, CRSwNP and CRSsNP patients presented an even larger difference in nNO levels. According to these sensitivity analyses, previous studies in patients with allergic rhinitis showed that intranasal steroids therapy significantly reduces NO production by blocking the transcription of the iNO-synthase gene [50,51]. Thus, the key role of osteomeatal complex obstruction in explaining the findings of our meta-analysis seems to be the most probable. It has been shown that although adult patients with CRSwNP have high levels of iNOS in the nasal epithelium due to inflammation of nasal and paranasal cavities, they exhibit reduced levels of nNO in comparison with subjects affected by uncomplicated allergic rhinitis [52]. Additionally, a higher degree of polyposis brings about lower nNO levels [52]. Accordingly, restoring patency of sinus ostia by endoscopic surgery has shown to be associated with a rapid increase in nNO levels [42,43]. Overall, these findings, confirming our results, suggest the hypothesis that the low nNO levels in patients with paranasal sinus inflammatory diseases are generated by mechanical obstruction of the draining ostia and by the negative pressure within the sinuses, resulting in a reduced transit of NO from sinuses to the nasal lumen, despite the increased NO synthesis due to inflammation.

Our findings are in line with this great amount of both in-vitro and clinical studies suggesting that measuring nNO can objectively reflect patency of sinus ostia in patients with chronic rhinosinusitis. Our systematic literature search confirms that many authors tried to evaluate the impact of CRSwNP and CRSsNP on nNO levels over time, with conflicting results. This may be due to the heterogeneous populations enrolled in each study, with a great amount of concomitant clinical confounding factors, such as asthma or the use of intranasal corticosteroids. Another important factor that may account for the different results observed across studies is the lack of a single standardized method for nNO assessment. Currently, two main approaches can be described: the first one (nasal aspiration) uses a single nostril during closure of the velum, while the second one (nasal exhalation) uses a tight facemask with a steady flow fixed in advance (similar to oral FeNO) [11]. Another technical aspect potentially and significantly affecting nNO levels is transnasal airflow [53]. With both methods, the nNO measurement involves the production of a stable airflow through the nasal cavity. This produces an increase in nNO levels followed by the achievement of a plateau [13], with the nNO value being inversely correlated with the transnasal airflow [54]. Obviously, the different populations enrolled in each study together with such differences among assessment techniques for nNO may account for the different results observed across studies over time. By using SMD instead of mean difference, we were able to (partially) overcome such heterogeneity among study results since SMD should be used when measures obtained with different methods are examined together [24]. Moreover, we evaluated the impact of such confounding factors by means of appropriate sensitivity and subgroup analyses and after implementing some meta-regression analyses. As a result, we found that the female gender might be associated with higher discriminating ability of nNO against the existence of polyps in subjects affected by chronic rhinosinusitis. Furthermore, our regression models indicated that an augmented aspiration flow is associated with an increased difference in nNO values among CRSwNP patients and controls (both CRSsNP and healthy subjects). Considering that all studies on exhalation method generally use a constant flow of 50 mL/s, the impact of transnasal airflow for studies on nasal exhalation was not evaluated in our meta-analysis. It should also be kept in mind that, when separately analyzing studies using different techniques, our results were confirmed only for the aspiration method. However, care should be used in interpreting the latter finding because of the limited number of studies (*n* = 5) evaluating nNO by nasal exhalation in our meta-analysis.

Further confirming the role of nNO as a biomarker of osteomeatal complex obstruction [55], our results suggest that higher aspiration flows should be used in order to differentiate the presence/absence of polyposis in subjects with nasal symptoms. Our findings, together with the great amount of literature data currently available, support the use of nNO dosage for the diagnosis and monitoring of nasal polyposis. However, if the absence of a standardized method along with the absence of comparable age-adjusted standard values may restrict the strength of nNO as a marker of sinus ostia patency, nNO assessment should be considered as an effective and non-invasive tool to monitor the effectiveness of surgical therapy. Despite the absence of reference values, a rapid increase of nNO levels in a patient after surgery could be considered as a sign of patency recovery after endoscopic surgery. However, there is still the need for large long-term prospective studies in order to define one single standardized method for nNO assessment and normal reference values in this and in other clinical settings.

Our study presents some possible limitations. Firstly, the data used for our meta-analysis enrolled patients with diverse demographic and clinical characteristics because of their different inclusion/exclusion criteria. Moreover, each study presented some missing information. Considering that meta-analyses are carried out on aggregate data, the multivariate procedure permitted the adjustment of some (but not all) probable confounders. Thus, though meta-regression analyses were able to refine analyses by evaluating the effect of most clinical and demographic variables on the results, carefulness is needed in the interpretation of the results.

Secondly, our results were affected by significantly high heterogeneity. Even though it was impossible to finally determine the origin of such heterogeneity, all findings were substantially demonstrated by proper sensitivity and subgroup analyses, and the influence of clinical and demographic variables on results was evaluated using meta-regression models. Furthermore, we excluded the presence of publication bias by using different methods, and in case of significant bias, our results were validated after trimming and imputing studies.

Finally, we have to consider that the lack of comparable age-adjusted standard values possibly will reduce the soundness of nNO as a marker of osteomeatal complex obstruction. Given the restricted number of studies (*n* = 3) assessing nNO diagnostic accuracy in patients affected by suspected polyposis, we were not able to carry out a meta-analysis of diagnostic accuracy at different cut-off values. This partially reduces the clinical application of our results.

All considered, our meta-analysis indicates that CRSwNP is meaningfully related to reduced nNO levels, especially when using higher nasal aspiration flows. Further and rigorously planned studies are required to define one single standardized method for nNO assessment and normal reference values in this and in other clinical settings.

## Figures and Tables

**Table 1:** Demographic and clinical data of patients with chronic rhinosinusitis with (CRSwNP) or without nasal polyps (CRSsNP) and healthy subjects in included studies.

Study		Pop (n)	Males (%)	Age (Years)	Smoking (%)	Atopy (%)	Asthma (%)	Intranasal CCS (%)	Lund–Mackay CT Score	SNOT-22 Score	FEV_1_ (% Predicted)	FEV_1_/FVC
CRSwNP vs. HEALTHY												
Arnal 1999—No allergy [28]	Pts	20	65.0	48.0	15.0	0	10.0	55.0	-	-	-	-
	Controls	42	52.4	42.0	0	0	N/A	N/A	N/A	N/A	-	-
Arnal 1999—Allergy [28]	Pts	7	71.4	42.0	15.0	100	71.4	14.3	-	-	-	-
	Controls	42	52.4	42.0	0	0	N/A	N/A	N/A	N/A	-	-
Asano 2018 [29]	Pts	11	-	-	0	100	100	0	-	-	-	-
	Controls	10	-	-	0	0	N/A	N/A	N/A	N/A	-	-
Bommarito 2008 [30]	Pts	30	63.3	52.0	0	57.0	-	0	13.5	-	-	-
	Controls	29	31.0	39.0	0	55.0	N/A	N/A	N/A	N/A	-	-
Frendø 2018 [31]	Pts	57	70.2	49.3	-	32.0	44.0	0	-	-	-	-
	Controls	30	36.7	34.5	-	0	N/A	N/A	N/A	N/A	-	-
Gilian 2002 * [32]	Pts	18	77.8	-	-	-	0	-	-	-	-	-
	Controls	21	33.3	-	-	-	N/A	N/A	N/A	N/A	-	-
Guilemany 2009 [33]	Pts	22	50.0	49.0	4.5	-	-	-	10.8	-	80.0	92.0
	Controls	20	50.0	59.0	0	-	N/A	N/A	N/A	N/A	90.0	96.0
Gupta 2013 [21]	Pts	24	-	54.5	-	-	-	-	-	28.2	-	-
	Controls	11	-	45.7	-	-	N/A	N/A	N/A	N/A	-	-
Heffler 2014 [34]	Pts	34	-	-	0	-	100	0	13.0	-	-	-
	Controls	40	25.0	48.7	0	20.0	N/A	N/A	N/A	N/A	97.2	82.3
Jeong 2014—No allergy [16]	Pts	30	53.3	33.4	-	0	0	0	14.6	-	-	-
	Controls	30	50.0	27.3	-	0	N/A	N/A	N/A	N/A	-	-
Jeong 2014—Allergy [16]	Pts	27	77.8	33.4	-	100	0	0	11.6	-	-	-
	Controls	30	50.0	27.3	-	0	N/A	N/A	N/A	N/A	-	-
Liu 2017 [35]	Pts	54	61.1	-	0	27.7	0	0	-	-	-	-
	Controls	20	85.0	35.5	0	0	N/A	N/A	N/A	N/A	-	-
Noda 2012—Medical group [36]	Pts	12	-	57.9	-	-	75.0	0	14.2	-	-	-
	Controls	32	-	50.0	-	-	N/A	N/A	N/A	N/A	-	-
Noda 2012—Surgical group [36]	Pts	24		56.2	-	-	58.3	0	16.9	-	-	-
	Controls	32	-	50.0	-	-	N/A	N/A	N/A	N/A	-	-
Torretta 2015 * [37]	Pts	37	-	-	-	-	-	-	-	-	-	-
	Controls	36	-	-	-	-	N/A	N/A	N/A	N/A	-	-
Tworek 2012 [38]	Pts	10	50.0	38.9	0	70.0	0	0	-	-	89.7	-
	Controls	10	60.0	41.0	0	60.0	N/A	N/A	N/A	N/A	92.2	-
Weschta 2008 [39]	Pts	6	-	-	-	-	-	0	-	-	-	-
	Controls	10	30.0	38.0	0	0	N/A	N/A	N/A	N/A	-	-
Williamson 2010 [40]	Pts	38	55.3	52.0	-	57.9	0	0	-	-	101.1	-
	Controls	41	46.3	27.0	-	0	N/A	N/A	N/A	N/A	98.4	-
CRSsNP vs. HEALTHY												
Alexandersson 2019 [27]	Pts	12	33.0	46.0	0	33.0	17.0	67.0	9.7	50.7	-	-
	Controls	12	42.0	35.0	0	50.0	N/A	N/A	N/A	N/A		
Arnal 1999 [28]	Pts	10	70.0	47.0	20.0	0	10.0	70.0	-	-	-	-
	Controls	42	52.4	42.0	0	0	N/A	N/A	N/A	N/A	-	-
Asano 2008 [29]	Pts	13	-	-	0	100	100	0	-	-	-	-
	Controls	10	-	-	0	0	N/A	N/A	N/A	N/A	-	-
Bommarito 2008 [30]	Pts	14	50.0	42.0	0	50.0	-	0	5.7	-	-	-
	Controls	29	31.0	39.0	0	55.0	N/A	N/A	N/A	N/A	-	-
Guilemany 2009 [33]	Pts	46	13.0	56.0	9.0	-	-	-	8.4	-	81.0	92.0
	Controls	20	50.0	59.0	0	-	N/A	N/A	N/A	N/A	90.0	96.0
Gupta 2013 [21]	Pts	31	-	52.3	-	-	-	-	-	25.2	-	-
	Controls	11	-	45.7	-	-	N/A	N/A	N/A	N/A	-	-
Heffler 2013 [34]	Pts	7	-	-	0	-	100	0	3.0	-	-	-
	Controls	40	25.0	48.7	0	20.0	N/A	N/A	N/A	N/A	97.2	82.3
Lindenberg 1997 [17]	Pts	12	50.0	46.8	25.0	22.7	-	0	-	-	-	-
	Controls	66	50.0	34.6	9.1	16.7	N/A	N/A	N/A	N/A	-	-
Liu 2017 [35]	Pts	34	79.4	-	0	38.2	0	0	-	-	-	-
	Controls	20	85.0	35.5	0	0	N/A	N/A	N/A	N/A	-	-
Weschta 2008 [39]	Pts	4	-	-	-	-	-	0			-	-
	Controls	10	30.0	38.0	0	0	N/A	N/A	N/A	N/A	-	-
CRSwNP vs. CRSsNP												
Arnal 1999 [28]	Pts	20	65.0	48.0	15.0	0	10.0	55.0	-	-	-	-
	Controls	10	70.0	47.0	20.0	0	10.0	70.0	-	-	-	-
Asano 2008 [29]	Pts	11	-	-	0	100	100	0	-	-	-	-
	Controls	13	-	-	0	100	100	0	-	-	-	-
Bae 2014 * [41]	Pts	16	-	-	-	-	-	-	-	-	-	-
	Controls	19	-	-	-	-	-	-	-	-	-	-
Bommarito 2008 [30]	Pts	30	63.3	52.0	0	57.0	-	0	13.5	-	-	-
	Controls	14	50.0	42.0	0	50.0	0	0	5,7	-	-	-
Fu 2015 [42]	Pts	53	67.9	44.5	17.0	18.9	20.8	-	16.4	42.7	-	-
	Controls	16	56.2	45.6	18.0	25.0	12.5	-	11.8	47.7	-	-
Fu 2017 [20]	Pts	12	45.9	50.0	33.3	41.7	0	-	9.8	39.3	-	-
	Controls	13	52.6	53.8	30.7	30.7	0	-	6.5	37.6	-	-
Guilemany 2009 [33]	Pts	22	50.0	49.0	4.5	-	-	-	10.8	-	80.0	92.0
	Controls	46	13.0	56.0	9.0	-	-	-	8.4	-	81.0	92.0
Gupta 2013 [21]	Pts	24	-	54.5	-	-	-	-	-	28.2	-	-
	Controls	31	-	52.3	-	-	-	-	-	25.2	-	-
Heffler 2013 [34]	Pts	34	-	-	0	-	100	0	13.0	-	-	-
	Controls	7	-	-	0	-	100	0	3.0	-	-	-
Lee 2015 [18]	Pts	33	-	-	-	-	-	100	-	-	-	-
	Controls	6	-	-	-	-	0	0	-	-	-	-
Liu 2017 [35]	Pts	54	61.1	-	0	27.7	0	0	-	-	-	-
	Controls	34	79.4	-	0	38.2	0	0	-	-	-	-
Ragab 2006—Medical group [43]	Pts	16	-	-	-	-	-	-	-	-	-	-
	Controls	29	-	-	-	-	0	-	-	-	-	-
Ragab 2006—Surgical group [43]	Pts	19	-	-	-	-	-	-	-	-	-	-
	Controls	25	-	-	-	-	0	-	-	-	-	-
Weschta 2008 [39]	Pts	6	-	-	-	-	-	0	-	-	-	-
	Controls	4	-	-	-	-	-	0	-	-	-	-
Yoshida 2019 [19]	Pts	22	-	-	-	-	-	0	-	-	-	-
	Controls	30	-	-	-	-	-	0	-	-	-	-

Pop: population; n: number; pts: patients; CCS: corticosteroids; CT: computed tomography; SNOT-22: Sino-Nasal Outcome Test-22; FEV_1_: forced expiratory volume in 1 second; FVC: forced vital capacity; N/A: not applicable. Continuous data are expressed as mean values, unless otherwise indicated. * Only abstract available.

**Table 2:** Sensitivity analyses. (Panel **A**): “high quality” studies (i.e., Newcastle–Ottawa Scale ≥ 6); (Panel **B**): exclusion of studies potentially reporting on the same population as other included studies; (Panel **C**): exclusion of studies reporting on patients in therapy with local steroids.

	N of Studies	N of Patients	Effect Size SMD (95% CI) in nNO
**“High Quality” Studies**			**Panel A**
CRSwNP vs. healthy	12	370 pts	SMD: −1.567 (−2.155, −0.978); *p* < 0.0001
	(14 data-sets)	295 controls	I^2^ = 90.5%; *p* <0.0001
CRSsNP vs. healthy	9	171 pts	SMD: −0.550 (−1.002, −0.097); *p* = 0.017
	(9 data-sets)	194 controls	I^2^ = 70.6%; *p* < 0.0001
CRSwNP vs. CRSsNP	13	356 pts	SMD: −1.565 (−2.174, −0.957); *p* < 0.0001
	(14 data-sets)	278 controls	I^2^ = 89.5%; *p* < 0.0001
**Exclusion of duplicate populations**			**Panel B**
CRSwNP vs. healthy	15	461 pts	SMD: −1.495 (−2.135, −0.854); *p* < 0.0001
	(18 data-sets)	384 controls	I^2^ = 93.9%; *p* < 0.0001
CRSsNP vs. healthy	10	183 pts	SMD: −0.696 (−1.189, −0.202); *p* = 0.006
	(10 data-sets)	260 controls	I^2^ = 78.2%; *p* < 0.0001
CRSwNP vs. CRSsNP	13	360 pts	SMD: −1.554 (−2.171, −0.936); *p* < 0.0001
	(14 data-sets)	284 controls	I^2^ = 90.0%; *p* < 0.0001
**Exclusion of patients receiving intranasal CCS**			**Panel C**
CRSwNP vs. healthy	10	333 pts	SMD: −1.254 (−1.974, −0.534); *p* = 0.001
	(12 data-sets)	318 controls	I^2^ = 93.3%; *p* < 0.0001
CRSsNP vs. healthy	5	72 pts	SMD: −0.456 (−1.278, 0.367); *p* =0.277
	(5 data-sets)	109 controls	I^2^ = 81.7%; *p* < 0.0001
CRSwNP vs. CRSsNP	6	157 pts	SMD: −1.953 (−2.588, −1.318); *p* < 0.0001
	(6 data-sets)	102 controls	I^2^ = 90.0%; *p* < 0.0001

N: number; SMD: standardized mean difference; 95% CI: 95% Confidence Intervals; nNO: nasal nitric oxide; CRSwNP: chronic rhinosinusitis with nasal polyps; CRSsNP: chronic rhinosinusitis without nasal polyps; CCS: corticosteroids. Significance was set at *p* < 0.05. Statistically significant results are shown in bold.

**Table 3:** Subgroup analyses. (Panel **A**): studies on nasal nitric oxide (nNO) measured by nasal aspiration method; (Panel **B**): studies on nNO measured by nasal exhalation method.

	N of Studies	N of Patients	Effect Size SMD (95% CI) in nNO
**Nasal Aspiration**			**Panel A**
CRSwNP vs. healthy	12	408 pts	SMD: −1.885 (−2.547, −1.223); *p* < 0.0001
	(14 data-sets)	332 controls	I^2^ = 93.0%; *p* < 0.0001
CRSsNP vs. healthy	8	166 pts	SMD: −0.862 (−1.384, −0.340); *p* = 0.001
	(8 data-sets)	240 controls	I^2^ = 78.4%; *p* < 0.0001
CRSwNP vs. CRSsNP	10	320 pts	SMD: −1.451 (−2.124, −0.779); *p* < 0.0001
	(11 data-sets)	231 controls	I^2^ = 90.3%; *p* < 0.0001
**Nasal exhalation**			**Panel B**
CRSwNP vs. healthy	3	53 pts	SMD: −0.022 (−1.067, 1.022); *p* = 0.966
	(4 data-sets)	52 controls	I^2^ = 86.2%; *p* < 0.0001
CRSsNP vs. healthy	2	17 pts	SMD: 0.163 (−0.512, 0.839); *p* = 0.636
	(2 data-sets)	20 controls	I^2^ = 0%; *p* = 0.461
CRSwNP vs. CRSsNP	4	52 pts	SMD: −1.448 (−3.018, 0.121); *p* = 0.071
	(4 data-sets)	66 controls	I^2^ = 92.0%; *p* < 0.0001

N: number; SMD: standardized mean difference; 95% CI: 95% Confidence Intervals; nNO: nasal nitric oxide; CRSwNP: chronic rhinosinusitis with nasal polyps; CRSsNP: chronic rhinosinusitis without nasal polyps. Significance was set at *p* < 0.05. Statistically significant results are shown in bold.

## Supplementary Materials

The following are available online at https://www.mdpi.com/2077-0383/9/1/200/s1, Table S1: Devices and methods for measurement of nasal nitric oxide (nNO) in included studies, Table S2: Assessment of quality of studies (Newcastle-Ottawa scale), Table S3: Meta-regression analyses. Impact of major clinical and demographic variables on nasal nitric oxide (nNO) in cases and control subjects; Figure S1: Funnel plot of effect size vs. precision (1/standard error) for studies evaluating nasal nitric oxide (nNO) in patients with chronic rhinosinusitis with nasal polyps (CRSwNP) and healthy controls., Figure S2: Funnel plot of effect size vs. precision (1/standard error) for studies evaluating nasal nitric oxide (nNO) in patients with chronic rhinosinusitis without nasal polyps (CRSsNP) and healthy controls, Figure S3: Funnel plot of effect size vs. precision (1/standard error) for studies evaluating nasal nitric oxide (nNO) in patients with chronic rhinosinusitis with nasal polyps (CRSwNP) and in control subjects with chronic rhinosinusitis without nasal polyps (CRSsNP).
