# Supplementary material for: Nasal Nitric Oxide in Chronic Rhinosinusitis with or without Nasal Polyps: A Systematic Review with Meta-Analysis
Source: J Clin Med. 2020 Jan 11;9(1):200. doi: 10.3390/jcm9010200 (PMC7020063; doi:10.3390/jcm9010200)
Supplement: Supplementary file 1 [file jcm-09-00200-s001.pdf]

## Supplementary online material

**Nasal nitric oxide in chronic rhinosinusitis with or without nasal polyps:**

**a systematic review with meta-analysis.**


Pasquale Ambrosino [1], Antonio Molino [2], Giorgio Alfredo Spedicato [3], Paolo Parrella [1], Roberto Formisano [1], Andrea Motta [4], Matteo Nicola Dario Di Minno [5], Mauro Maniscalco [1]

[1]Cardio-pulmonary Rehabilitation Dept, Istituti Clinici Scientifici Maugeri IRCCS, Telese Terme, Italy;
[2]Respiratory Division, Department of Respiratory Medicine, Federico II University, Naples, Italy.
[3]University of Bologna, Bologna, Italy
[4]Institute of Biomolecular Chemistry, National Research Council, 80078 Pozzuoli (Naples), Italy.
[5]Department of Translational Medical Sciences, Federico II University, Naples, Italy;


Table of contents:


| | |
|---|---|
| Supplemental Table 1 | Devices and methods for measurement of nasal nitric oxide (nNO) in included studies. |
| Supplemental Table 2 | Assessment of quality of studies (Newcastle-Ottawa scale). |
| Supplemental Table 3 | Meta-regression analyses. Impact of major clinical and demographic variables on nasal nitric oxide (nNO) in cases and control subjects. |
| Supplemental Figure 1 | Funnel plot of effect size vs. precision (1/standard error) for studies evaluating nasal nitric oxide (nNO) in patients with chronic rhinosinusitis with nasal polyps (CRSwNP) and healthy controls. |
| Supplemental Figure 2 | Funnel plot of effect size vs. precision (1/standard error) for studies evaluating nasal nitric oxide (nNO) in patients with chronic rhinosinusitis without nasal polyps (CRSsNP) and healthy controls. |
| Supplemental Figure 3 | Funnel plot of effect size vs. precision (1/standard error) for studies evaluating nasal nitric oxide (nNO) in patients with chronic rhinosinusitis with nasal polyps (CRSwNP) and in control subjects with chronic rhinosinusitis without nasal polyps (CRSsNP). |

**Supplemental Table 1. Devices and methods for measurement of nasal nitric oxide (nNO) in included studies.**

| Study | Analyzer | Technique | Transnasal airflow | Device | Equipment |
|---|---|---|---|---|---|
| Alexandersson 2019 | Chemiluminescence | Aspiration during breath hold | 2000 ml/min | Nasal olive | ▪ NIOX®, Aerocrine AB (Solna,Sweden) |
| Arnal 1999 | Chemiluminescence | Aspiration during tidal breath | 700 ml/min | Nasal olive | ▪ NO analyser, Cosma (Igny, France) |
| Asano 2008 | Chemiluminescence | Nasal exhalation | 3000 ml/min | Nasal olive | ▪ Sievers NOA-280i®, GE Analytical Instruments (Boulder, USA) |
| Bae 2014 | Electrochemical | Nasal exhalation | - | - | ▪ NObreath®, Bedfont Scientific (Rochester, UK) |
| Bommarito 2008 | Chemiluminescence | Aspiration during breath hold | 300 ml/min | Nasal olive | ▪ NIOX®, Aerocrine AB (Solna,Sweden) |
| Frendø 2018 | Electrochemical | Aspiration during exhalation against resistance | 300 ml/min | Nasal olive | ▪ NIOX MINO Airway Inflammation Monitor®, Aerocrine AB (Solna,Sweden) |
| Fu 2015 | Electrochemical | Aspiration during tidal breath | 300 ml/min | Nasal olive | ▪ NIOX MINO Airway Inflammation Monitor®, Aerocrine AB (Solna,Sweden) |
| Fu 2017 | Electrochemical | Aspiration during tidal breath | 300 ml/min | Nasal olive | ▪ NIOX MINO Airway Inflammation Monitor®, Aerocrine AB (Solna,Sweden) |
| Gilain 2002 | Chemiluminescence | Aspiration during exhalation against resistance | 3000 ml/min | Nasal olive | ▪ Sievers NOA-280i®, GE Analytical Instruments (Boulder, USA) |
| Guilemany 2009 | Chemiluminescence | Aspiration during breath hold | 3000 ml/min | Nasal olive | ▪ SIR System N6008 NO tracer®, SIR (Madrid, Spain) |
| Gupta 2013 | Electrochemical | - | 300 ml/min | Nasal olive | ▪ NIOX MINO Airway Inflammation Monitor®, Aerocrine AB (Solna,Sweden) |
| Heffler 2013 | Chemiluminescence | Aspiration during breath hold | 300 ml/min | Nasal olive | ▪ NIOX®, Aerocrine AB (Solna,Sweden) |
| Jeong 2014 | Chemiluminescence | Aspiration during exhalation against resistance | 700 ml/min | Nasal olive | ▪ Sievers NOA-280i®, GE Analytical Instruments (Boulder, USA) |
| Lee 2015 | Chemiluminescence | Aspiration during exhalation against resistance | 300 ml/min | Nasal olive | ▪ Eco Medics CLD 88sp NO Analyzer®, Eco Physics Inc. (Ann Arbor, USA) |
| Lindenberg 1997 | Chemiluminescence | Aspiration during tidal breath | 660 ml/min | Nasal olive | ▪ CLD 700 AL Med®, Ecophysics (Durnten, Switzerland) |
| Liu 2017 | Electrochemical | Aspiration during exhalation against resistance | 2500-3000 ml/min | Nasal olive | ▪ NIOX MINO Airway Inflammation Monitor®, Aerocrine AB (Solna,Sweden) |
| Noda 2012 | Electrochemical | Nasal exhalation | 3000 ml/min | Nasal olive | ▪ NObreath®, Bedfont Scientific (Rochester, UK) |
| Ragab 2006 | Chemiluminescence | Aspiration during breath hold | 250 ml/min | Nasal olive | ▪ LR 2000®, Logan Sinclair (Rochester, UK) |

| | | | | | |
|---|---|---|---|---|---|
| Torretta 2015 | - | - | - | - | ▪ - |
| Tworek 2012 | Electrochemical | - | - | Nasal olive | ▪ ExpAir®, Medisoft (Dinant, Belgium) |
| Weschta 2008 | Electrochemical | Nasal exhalation | 3000 ml/min | Nasal mask | ▪ NIOX MINO Airway Inflammation Monitor®, Aerocrine AB (Solna,Sweden) |
| Williamson 2010 | Chemiluminescence | Aspiration during exhalation against resistance | - | Nasal olive | ▪ NIOX®, Aerocrine AB (Solna,Sweden) |
| Yoshida 2019 | Chemiluminescence | Nasal exhalation | 3000 ml/min | Nasal olive | ▪ Sievers NOA-280i®, GE Analytical Instruments (Boulder, USA) |

**Supplemental Table S2. Assessment of quality of studies (Newcastle-Ottawa scale).**

| Study | Adequate case definition | Representativeness of the cases | Selection of controls | Definition of controls | Comparability of cases and controls on the basis of the design or analysis* | Ascertainment of exposure | Same method of ascertainment for cases and controls | Non-response rate | Quality |
|---|---|---|---|---|---|---|---|---|---|
| Alexandersson 2019 | ✶ | ✶ | - | ✶ | - | ✶ | ✶ | ✶ | 6 |
| Arnal 1999 | ✶ | - | - | ✶ | ✶ | ✶ | ✶ | ✶ | 6 |
| Asano 2008 | ✶ | ✶ | - | ✶ | - | ✶ | ✶ | ✶ | 6 |
| Bae 2014** | - | - | - | - | - | - | - | - | NA |
| Bommarito 2008 | ✶ | ✶ | - | ✶ | - | ✶ | ✶ | ✶ | 6 |
| Frendø 2018 | ✶ | ✶ | ✶ | ✶ | - | ✶ | ✶ | ✶ | 7 |
| Fu 2015 | ✶ | ✶ | ✶ | ✶ | ✶ | ✶ | ✶ | ✶ | 8 |
| Fu 2017 | ✶ | ✶ | ✶ | ✶ | ✶ | ✶ | ✶ | ✶ | 8 |
| Gilain 2002** | - | - | - | - | - | - | - | - | NA |
| Guilemany 2009 | ✶ | ✶ | ✶ | ✶ | - | ✶ | ✶ | ✶ | 7 |
| Gupta 2013 | ✶ | ✶ | - | ✶ | - | ✶ | ✶ | ✶ | 6 |
| Heffler 2013 | ✶ | ✶ | - | ✶ | - | ✶ | ✶ | ✶ | 6 |
| Jeong 2014 | ✶ | - | - | ✶ | ✶ | ✶ | ✶ | ✶ | 6 |
| Lee 2015 | ✶ | ✶ | ✶ | ✶ | - | ✶ | ✶ | ✶ | 7 |
| Lindenberg 1997 | ✶ | - | - | ✶ | - | ✶ | ✶ | ✶ | 5 |
| Liu 2017 | ✶ | ✶ | - | ✶ | - | ✶ | ✶ | ✶ | 6 |
| Noda 2012 | ✶ | - | - | ✶ | - | ✶ | ✶ | ✶ | 5 |
| Ragab 2006 | ✶ | ✶ | ✶ | ✶ | - | ✶ | ✶ | ✶ | 7 |
| Torretta 2015** | - | - | - | - | - | - | - | - | NA |
| Tworek 2012 | ✶ | - | - | ✶ | ✶✶ | ✶ | ✶ | ✶ | 7 |
| Weschta 2008 | ✶ | ✶ | ✶ | ✶ | - | ✶ | ✶ | ✶ | 7 |

| | | | | | | | | | |
|---|---|---|---|---|---|---|---|---|---|
| Williamson 2010 | ✶ | ✶ | - | ✶ | ✶✶ | ✶ | ✶ | ✶ | 8 |
| Yoshida 2019 | ✶ | ✶ | - | ✶ | - | ✶ | ✶ | ✶ | 6 |

**NA: not assessed.**

*A maximum of 2 stars can be allotted in this category: ✶if enrolling controls matched to cases for age and sex; ✶✶ if enrolling controls matched to cases for any additional factor.

**Only abstract available.

**Supplemental Table 3. Meta-regression analyses. Impact of major clinical and demographic variables on nasal nitric oxide (nNO) in cases and control subjects.**

| | Independent variable | nNO |
|---|---|---|
| **CRSwNP vs. HEALTHY** | % of males | Z-score: 0.115, P=0.908 |
| | Age | Z-score: 1.058, P=0.290 |
| | Smoking | Z-score: 1.707, P=0.088 |
| | Atopy | Z-score: 0.178, P=0.858 |
| | Asthma | Z-score: 1.455, P=0.146 |
| | Lund-Mackay CT score | Z-score: 0.596, P=0.551 |
| | SNOT-22 score | N/A |
| | $FEV_1$ (% predicted) | Z-score: 1.401, P=0.161 |
| | FVC (% predicted) | N/A |
| | $FEV_1$/FVC | N/A |
| | Aspiration flow | **Z-score: -4.379, P<0.0001** |
| **CRSsNP vs. HEALTHY** | % of males | Z-score: -0.951, P=0.342 |
| | Age | Z-score: 0.484, P=0.629 |
| | Smoking | N/A |
| | Atopy | Z-score: 1.011, P=0.312 |
| | Asthma | Z-score: 1.975, P=0.050 |
| | Lund-Mackay CT score | **Z-score: -2.123, P=0.034** |
| | SNOT-22 score | Z-score: -0.455, P=0.649 |
| | $FEV_1$ (% predicted) | N/A |
| | FVC (% predicted) | N/A |
| | $FEV_1$/FVC | N/A |
| | Aspiration flow | Z-score: -0.473, P=0.636 |
| **CRSwNP vs. CRSsNP** | % of males | **Z-score: 2.631, P=0.009** |
| | Age | Z-score: -0.831, P=0.406 |
| | Smoking | Z-score: 1.576, P=0.115 |
| | Atopy | Z-score: -0.723, P=0.469 |
| | Asthma | Z-score: -1.437, P=0.151 |
| | Lund-Mackay CT score | Z-score: 0.515, P=0.607 |
| | SNOT-22 score | Z-score: -0.613, P=0.540 |
| | $FEV_1$ (% predicted) | N/A |
| | FVC (% predicted) | N/A |
| | $FEV_1$/FVC | N/A |
| | Aspiration flow | **Z-score: -4.100, P<0.0001** |

**CRSwNP: chronic rhinosinusitis with nasal polyps; CRSsNP: chronic rhinosinusitis without nasal polyps; CT: computed tomography; SNOT-22: Sino-Nasal Outcome Test-22; $FEV_1$: forced expiratory volume in 1 second; FVC: forced vital capacity; N/A: not assessed because of the limited number of studies reporting this covariate.**
**The overall effect was tested using Z-scores and significance was set at *P* <0.05. Statistically significant results are shown in bold.**

**Supplemental Figure 1. Funnel plot of effect size vs. precision (1/standard error) for studies evaluating nasal nitric oxide (nNO) in patients with chronic rhinosinusitis with nasal polyps (CRSwNP) and healthy controls.**

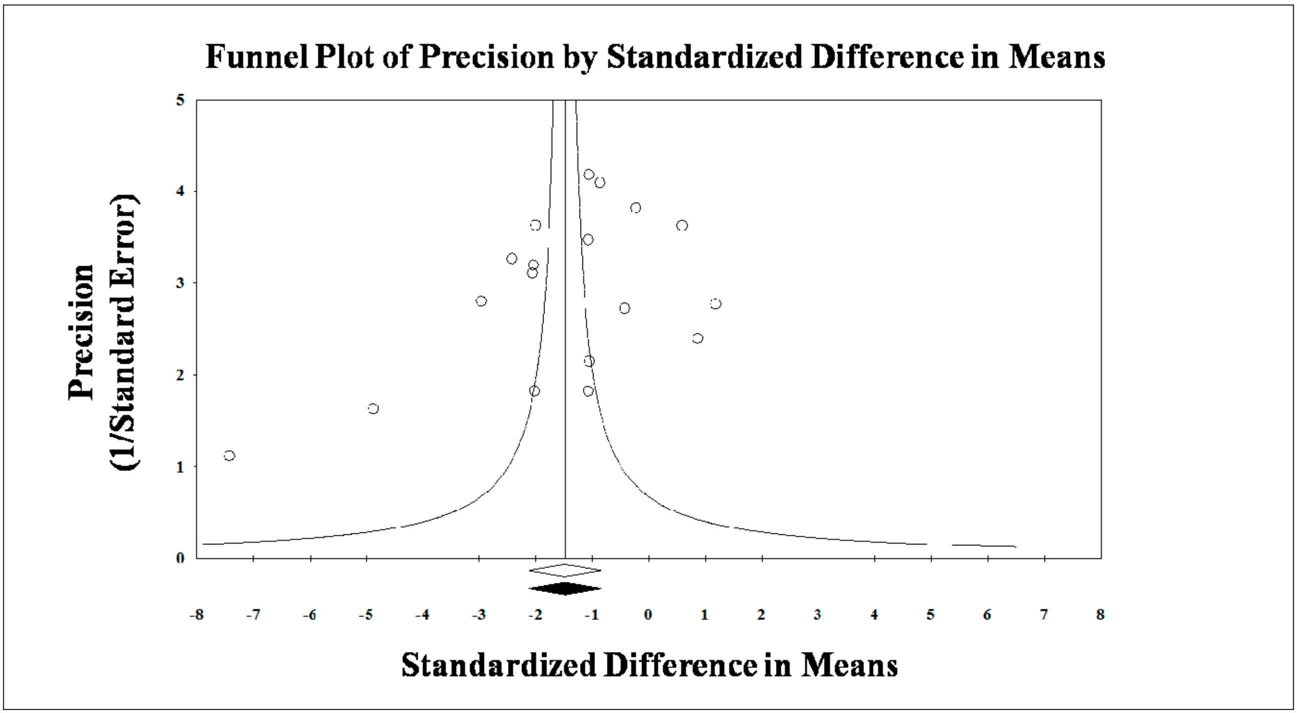


**Observed studies and effect size are represented by empty circles and empty square. Imputed studies and adjusted effect size are represented by black circles and black square.**

| | | |
|---|---|---|
| **Egger's regression intercept** | Intercept | -5.24799 |
| | Standard error | 3.13557 |
| | 95% lower limit | -11.89511 |
| | 95% upper limit | 1.39913 |
| | t-value | 1.67369 |
| | P value | 0.11362 |
| **Begg and Mazumdar** | **Kendall's S statistic (P-Q)** | -39.00000 |
| | **Kendall's tau without continuity correction** | |
| | tau | -0.25490 |
| | Z-value for tau | 1.47723 |
| | P-value | 0.13961 |
| | **Kendall's tau with continuity correction** | |
| | tau | -0.24837 |
| | Z-value for tau | 1.43935 |
| | P-value | 0.15005 |

| **Duvall and Tweedie's trim and fill** | Studies trimmed | Point estimate | Lower limit | Upper limit | **Q value** |
|---|---|---|---|---|---|
| Observed values | - | -1.495 | -2.135 | -0.854 | 270.216 |
| Adjusted values (on the left) | 0 | -1.495 | -2.135 | -0.854 | 270.216 |
| Adjusted values (on the right) | 0 | -1.495 | -2.135 | -0.854 | 270.216 |

**Supplemental Figure 2. Funnel plot of effect size vs. precision (1/standard error) for studies evaluating nasal nitric oxide (nNO) in patients with chronic rhinosinusitis without nasal polyps (CRSsNP) and healthy controls.**

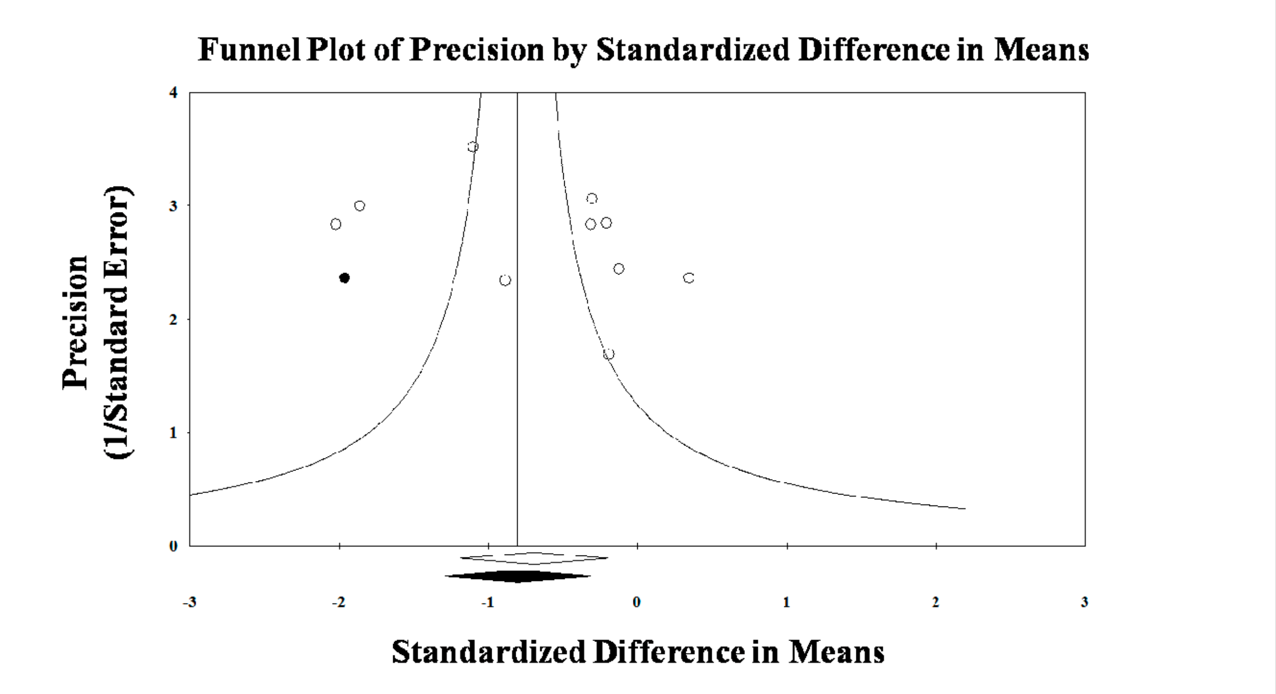


**Observed studies and effect size are represented by empty circles and empty square. Imputed studies and adjusted effect size are represented by black circles and black square.**

| | | |
|---|---|---|
| **Egger's regression intercept** | Intercept | 4.45525 |
| | Standard error | 3.79837 |
| | 95% lower limit | -4.30382 |
| | 95% upper limit | 13.21431 |
| | t-value | 1.17294 |
| | P value | 0.27456 |
| **Begg and Mazumdar** | **Kendall's S statistic (P-Q)** | 7.00000 |
| | **Kendall's tau without continuity correction** | |
| | tau | 0.15556 |
| | Z-value for tau | 0.62610 |
| | P-value | 0.53125 |
| | **Kendall's tau with continuity correction** | |
| | tau | 0.13333 |
| | Z-value for tau | 0.53666 |
| | P-value | 0.59151 |

| **Duvall and Tweedie's trim and fill** | Studies trimmed | Point estimate | Lower limit | Upper limit | **Q value** |
|---|---|---|---|---|---|
| Observed values | - | -0.696 | -1.189 | -0.202 | 41.214 |
| Adjusted values (on the left) | 1 | -0.804 | -1.294 | -0.315 | 48.502 |
| Adjusted values (on the right) | 0 | -0.696 | -1.189 | -0.202 | 41.214 |

**Supplemental Figure 3. Funnel plot of effect size vs. precision (1/standard error) for studies evaluating nasal nitric oxide (nNO) in patients with chronic rhinosinusitis with nasal polyps (CRSwNP) and in control subjects with chronic rhinosinusitis without nasal polyps (CRSsNP).**

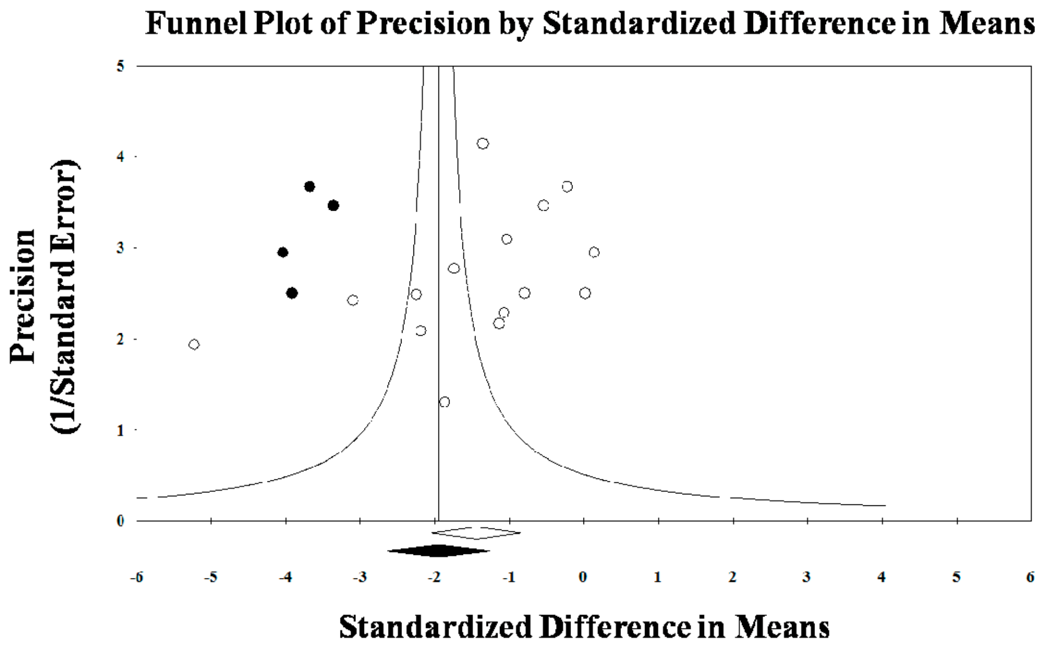


**Observed studies and effect size are represented by empty circles and empty square. Imputed studies and adjusted effect size are represented by black circles and black square.**

| | | |
|---|---|---|
| **Egger's regression intercept** | Intercept | -5.51859 |
| | Standard error | 2.94419 |
| | 95% lower limit | -11.87912 |
| | 95% upper limit | 0.84193 |
| | t-value | 1.87440 |
| | P value | 0.08352 |
| **Begg and Mazumdar** | **Kendall's S statistic (P-Q)** | -47.0000 |
| | **Kendall's tau without continuity correction** | |
| | tau | -0.44762 |
| | Z-value for tau | 2.32590 |
| | P-value | 0.02002 |
| | **Kendall's tau with continuity correction** | |
| | tau | -0.43810 |
| | Z-value for tau | 2.27641 |
| | P-value | 0.02282 |

| **Duvall and Tweedie's trim and fill** | Studies trimmed | Point estimate | Lower limit | Upper limit | **Q value** |
|---|---|---|---|---|---|
| Observed values | - | -1.448 | -2.046 | -0.850 | 140.342 |
| Adjusted values (on the left) | 4 | -1.955 | -2.264 | -1.268 | 328.771 |
| Adjusted values (on the right) | 0 | -1.448 | -2.046 | -0.850 | 140.342 |
